# Review on the Anti-Hyperglycemic Potential of *Psidium guajava* and *Seriphium plumosum* L.

**DOI:** 10.3390/plants13121608

**Published:** 2024-06-11

**Authors:** Nokukhanya Thembane, Sphamandla Hlatshwayo, Mlungisi Ngcobo, Phikelelani Ngubane, Nceba Gqaleni

**Affiliations:** 1 Department of Biomedical Sciences, Mangosuthu University of Technology, Durban 4026, South Africa; 2 Traditional Medicine Laboratory, University of KwaZulu-Natal, Durban 4041, South Africangcobom3@ukzn.ac.za (M.N.); gqalenin@ukzn.ac.za (N.G.); 3 Discipline of Medical Microbiology, University of KwaZulu-Natal, Durban 4041, South Africa; ngubanep1@ukzn.ac.za; 4 Africa Health Research Institute, Durban 4013, South Africa

**Keywords:** diabetes mellitus, type 2 diabetes mellitus, medicinal plants, phytochemicals, African traditional medicine, *Psidium guajava*, *Seriphium plumosum*

## Abstract

The treatment and management of diabetes mellitus (DM) with conventional therapies, such as insulin injections and oral hypoglycemic agents, present significant challenges due to their side effects and burdensome administration. Therapies often manage symptoms rather than addressing insulin regulation, akin to medications like thiazolidinediones and glinides, which resemble many medicinal plants. Medicinal plants offer potential alternative treatments due to bioactive compounds targeting diabetes causes. We aimed to explore the antidiabetic potential of two medicinal plants, *Psidium guajava* and *Seriphium plumosum* L., by investigating their phytochemical constituents, medicinal uses, pharmacological actions, and mechanisms. This review followed specific guidelines and searched databases including PubMed, Scopus, ScienceDirect, and Web of Science for studies on medicinal plants and DM. Eligible studies underwent quality assessment and were categorized based on their design and interventions for data synthesis. This review identified the phytochemical constituents in *Psidium guajava* and *Seriphium plumosum* L., including tannins, flavonoids, phenols, and steroids, exerting antidiabetic effects through various mechanisms like antioxidant activity, anti-inflammatory effects, stimulation of insulin secretion, glucose regulation, and inhibition of carbohydrate-digesting enzymes. *Psidium guajava* and *Seriphium plumosum* L. exhibit promising antidiabetic potential, offering alternative approaches to diabetes management. Polyherbalism, combining multiple plant extracts, may enhance therapeutic efficacy in diabetes treatment. Comprehensive research is needed to explore the combined therapeutic effects of these plants and develop more effective antidiabetic treatments. This review highlights the importance of harnessing natural resources to combat the global burden of DM. Further research is warranted to fully explore the combined therapeutic effects of these plants and develop novel treatments.

## 1. Introduction

Diabetes mellitus, a complex metabolic disorder characterized by impaired insulin production or function, poses significant challenges in its treatment [1]. The current therapeutic approaches primarily involve the use of insulin injections and oral hypoglycemic agents to control serum glucose concentration [1]. However, these treatments have limitations that necessitate the exploration of alternative and more effective options.

For instance, insulin injections can be burdensome for patients, requiring tolerance to needles, regular monitoring, and precise dosing [2]. Additionally, chronic insulin administration can lead to hypoglycemia, weight gain, and injection site reactions [3]. Oral hypoglycemic agents, although convenient, may cause gastrointestinal side effects and can be contraindicated in certain individuals [4]. Moreover, both approaches mainly focus on managing symptoms rather than addressing the underlying causes of DM.

Therefore, there is a pressing need for innovative alternative treatments that can achieve glycemic control, improve patient compliance, and address the pathophysiological mechanisms driving the onset of the disease. Exploring the therapeutic potential of medicinal plants for DM treatment offers a promising avenue for addressing the root causes of the disease. These plants possess bioactive compounds that can promote pancreatic beta cell regeneration, enhance insulin sensitivity, and optimize glucose metabolism [5].

However, challenges such as standardization, identification of active compounds, optimal dosage determination, safety considerations, and potential interactions with other medications need further investigation [6]. By overcoming these limitations through rigorous research, medicinal plants have the potential to reform DM treatment, providing individuals with favorable and healthier outcomes [5,7]

This narrative literature review is prompted by the existing anecdotal evidence of the combined use of the selected medicinal plants in diabetes management. It provides insights into the antidiabetic potential of *Psidium guajava* (*P. guajava*) and *Seriphium plumosum* L. (*S. plumosum*), highlighting their phytochemical constituents, medicinal uses, pharmacological actions, and mechanisms of action. Emphasizing the need for further research to explore their combined therapeutic effects, this review underscores the significance of exploring alternative antidiabetic treatments.

By pursuing alternative treatment options, such as those rooted in ethnomedicine, we can strive for better outcomes and improved quality of life for individuals living with type 2 diabetes mellitus (T2DM). Ethnomedicine, studying traditional medical practices globally, offers insights into ancient healing traditions. For instance, African traditional medicine uses indigenous plants for health, focusing on holistic well-being. The antidiabetic potential of plants like *P. guajava* and *S. plumosum* gains significance, bridging traditional and modern medicine.

## 2. Literature Search Strategy

The literature search encompassed electronic databases such as PubMed, Scopus, ScienceDirect, and Web of Science, covering studies up to April 2024. Specific keywords related to medicinal plants, herbal medicine, phytotherapy, traditional medicine, *Psidium guajava*, *Seriphium plumosum* L., diabetes, hyperglycemia, glucose metabolism, and insulin resistance were used to retrieve relevant articles. Studies were screened independently for relevance to anti-hyperglycemic potential in humans, animals, or in vitro models. After full-text retrieval and exclusion of duplicates, insufficient data, and non-English publications, the search identified 308 studies for possible inclusion, with 74 studies meeting the inclusion criteria included in the review.

### 2.1. The Antidiabetic Potential of Seriphium plumosum *L.* (S. plumosum)

*Seriphium plumosum* L. belongs to the Asteraceae family and is also known as silver stoebe in English and slangbos, vaalbossie, and Khoi-kooigoed in Afrikaans [6]. The Seriphium genus comprises a total of 36 species, with 2 species naturally occurring in Madagascar and 34 species found in South Africa [7]. Among these, *S. plumosum* is identified as the most vigorous and rapidly growing species [7].

#### 2.1.1. Description of *Seriphium plumosum* L.

**Figure 1:**
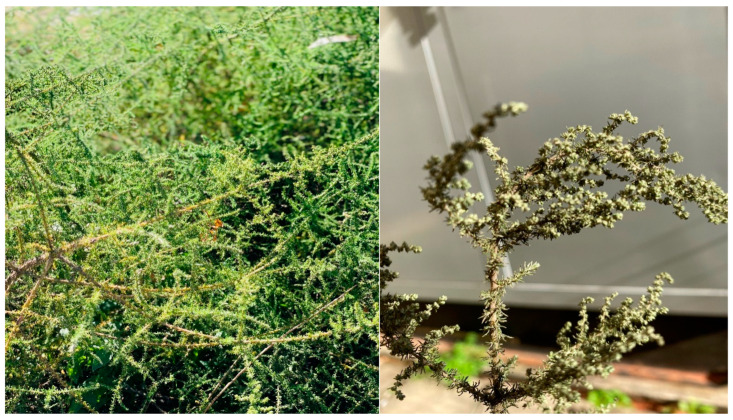
Images of *S. plumosum* L., locally known as *unwele* in IsiZulu (By N. Thembane, in KwaZulu Natal, South Africa—17 April 2023).

The botanical name Seriphium is derived from the word “seriph”, which denotes a stroke or line of letters. The term “plumosum” in the name *S. plumosum* means feathery, indicating the plant’s characteristic of having feather-like or feathery features [6]. The image showing the physical features of the plant is depicted in Figure 1.

*S. plumosum* is characterized by densely branched shrublet with a heath-like appearance, emerging from the ground [8]. This perennial woody dwarf shrub can grow up to a maximum of a single meter [9]. The stems of *S. plumosum* are adorned with small, feathery, and grayish leaves [8]. The flowerheads are grouped in small clusters towards the ends of the main shoots, forming a spike-like inflorescence. Pale brown bracts surround the purple disc florets, giving the spikes a golden appearance. Flowering occurs in autumn/winter, from April to June, but also in spring [6].

#### 2.1.2. Distribution of *Seriphium plumosum* L.

The *Seriphium plumosum* L. species is primarily distributed across several provinces in South Africa, including Limpopo, Northwest, Free State, Eastern Cape, Mpumalanga, Gauteng, and certain parts of KwaZulu-Natal. In natural habitats, *S. plumosum* exhibits morphological variations, including assorted color forms. The silver-grey form is commonly observed at higher elevations and displays slightly thicker stems compared to other variants [8]. The latest reports highlight the growing concern over *S. plumosum*’s invasive nature in South African grasslands [10]. The encroachment of *S. plumosum* in South Africa has led to the transformation of large grassland expanses into shrubland–grassland ecosystems of lower productivity [11]. *S. plumosum* is a serious threat to the sustainable production of grasslands in the Eastern Cape, Free State, Mpumalanga, North-West, and Gauteng provinces of South Africa [8]. The invasive nature of this encroaching shrub has posed a significant risk to the survival of approximately eleven million hectares of palatable grassland in South Africa, as reported by [11]. Previously, the expansion of *S. plumosum* was said to be attributed to the absence of controlled burning and selective grazing, particularly by sheep, following the establishment of livestock farming in these regions [8]. As a consequence, significant economic investments are made annually in countries like South Africa and Lesotho to control the spread of this species using chemical methods [12,13].

#### 2.1.3. Phytochemical Constituents of *Seriphium plumosum* L.

A qualitative analysis conducted in 2017 on the crude plant extracts of *S. plumosum* L. reported that tannins, *flavonoids*, phenols, and steroids were present in all the extracts [14]. Coumarins, on the other hand, were present in the methanolic and acetone extracts only. These phytochemicals have been studied for their potential role in the management of DM. While they may not directly cure T2DM, they have been shown to possess properties that facilitate management of the condition [14].

Table 1 highlights the available literature on how *S. plumosum* is used by the Indigenous people of Lesotho and South Africa. *S. plumosum* has various uses, as it is utilized in making household brooms, acts as an insect repellent, and serves as a nesting material for animals. Additionally, it is traditionally used by Indigenous people for diabetes, cardiac conditions, and epilepsy.

Table 2 highlights the various pathways through which phytochemical constituents exert their antidiabetic effects. These pathways include antioxidant activity, anti-inflammatory effects, insulin secretion and glucose regulation, inhibition of carbohydrate-digesting enzymes, improvement of insulin sensitivity, and modulation of lipid metabolism. Each pathway is associated with specific phytochemical constituents and their referenced studies.

The plant has antioxidant activity, which helps to protect against the development and the progression of DM complications by lowering oxidative stress [17]. These compounds are also anti-inflammatory and insulin sensitivity modulators, arresting the development of insulin resistance and DM [19]. Some phytochemicals increase insulin secretion and glucose absorption, resulting in better glucose control [20]. Certain phenolic substances inhibit carbohydrate-digesting enzymes, resulting in reduced intestinal glucose absorption [21]. Flavonoids promote insulin sensitivity, allowing for more efficient glucose metabolism [22]. Furthermore, plant steroids facilitate lipid metabolism, lowering cholesterol concentration and lowering the risk of cardiovascular complications. Understanding these mechanisms can guide the development of new treatments for DM [23]. However, only a single biomedical study explored its potential in managing DM. The study reported that the crude extracts of *S. plumosum* possessed tannins, steroids, coumarins, flavonoids, and phenols as phytochemical constituents [14]. Therefore, further research is needed to explore its diverse medicinal properties fully, specifically for its potential in DM management. *S. plumosum* has proven hypoglycemic properties in vitro, suggesting its potential as a natural remedy for DM [14]. Given the rising global prevalence of DM and the need for effective and sustainable treatments, investigating the medicinal properties of *S. plumosum* holds promise for future research and development of alternative therapeutic options.

## 3. The Antidiabetic Potential of *Psidium guajava*

*Psidium guajava* (*P. guajava*), commonly known as guava, is a member of the Myrtaceae family and stands out as a prominent fruit-bearing tree in South Africa. *P. guajava* is a shrub or small tree, usually not more than 4 m [24]. In South Africa, it is colloquially referred to as “guava” in English and “ugwava” in IsiZulu [25]. The *Psidium* genus includes about 150 species, with *P. guajava* being one of the most widely cultivated and appreciated for its prolific fruit production [26].

### 3.1. Description of P. guajava

**Figure 2:**
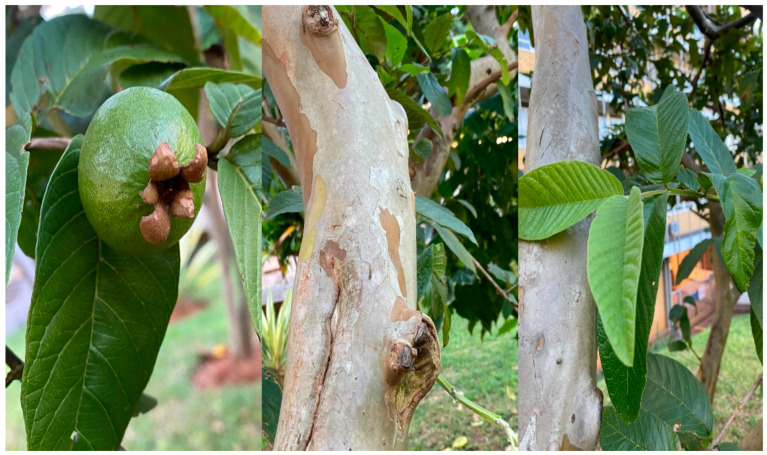
The unripe fruit, stem, and leaves of *Psidium guajava*, *ugwava* in IsiZulu (by N. Thembane in KwaZulu Natal, South Africa—17 April 2023).

Guava, scientifically identified as *P. guajava*, thrives in a diverse range of climates, showing adaptability to tropical and subtropical regions [27]. The botanical name, *P. guajava*, encapsulates the essence of this fruit-bearing plant. The bark peels off in flakes, revealing the characteristically smooth trunk [28], as depicted in Figure 2. When the foliage is observed, the plant has large leaves formed opposite each other in pairs with prominent veins, particularly on the lower side [29]. As the guava plant enters its flowering stage, it reveals delicate and fragrant white flowers, often found either individually or clustered in the leaf axils [30]. Flowering precedes the development of the guava fruits. While guava plants are known for year-round fruiting, peak flowering and fruit-bearing seasons may vary across varieties and local climates. In some places, guava flowers mostly appear in autumn and winter, from April to June, but blossoms may be observed on the plant in spring. Classified as an angiosperm, the medicinal plant features white flowers measuring approximately 25 mm in diameter, with many stamens typically blooming in early summer. Following the flowering stage, the plant produces rounded or pear-shaped fruits that range from 4 to 12 cm in size [31].

### 3.2. Distribution of P. guajava

Historically, *P. guajava* has been cultivated for commercialization in South Africa [32]. Nowadays, it is also found in Florida, Hawaii, Egypt, Brazil, the West Indies, Colombia, India, China, and Western nations due to its medical use [33]. The plant grows in tropic and subtropical climates [34]. In South Africa, this plant is found in the warm subtropical areas of KwaZulu Natal, Mpumalanga, and Northern Province [35]. *P. guajava* adapts to diverse altitudinal ranges and habitats, including tropical rainforests, riparian zones, and disturbed areas [34]. The plant adapts to temperature ranges between 15 and 30 °C. Its resilience to various climates and ecosystems contributes to its ecological versatility [34]. The plant holds cultural importance for its use in traditional cuisines and medicinal practices, while also playing a vital role in commercial agriculture, contributing to local economies through the sale of its fruits and derived products.

### 3.3. Phytochemical Constituents of P. guajava

*P. guajava* has been endowed by nature with a plethora of phytochemicals. Guava leaves are high in phenolic compounds, quercetin, avicularin, apigenin, guaijaverin, kaempferol, hyperin, myricetin, gallic acid, catechin, epicatechin, chlorogenic acid, epigallocatechin gallate, caffeic acid, isoflavonoids, catechin, rutin, naringenin, and kaempferol [36]. Ascorbic acid and carotenoids (lycopene, carotene, and cryptoxanthin) are abundant in the pulp [37]. Glycosydes, carotenoids, and phenolic compounds are found in the seeds, skin, and bark [38]. These compounds exhibit hepatoprotective, antioxidant, anti-inflammatory, antispasmodic, anti-cancer, antimicrobial, anti-hyperglycemic, analgesic, endothelial progenitor cell, anti-diarrheal, and anti-diarrhea properties [39].

### 3.4. Pharmacological Actions of P. guajava

Apart from the edible fruit, the various parts of guava have varied applications in medical health, namely as a remedy for mild to severe diarrhea, gastro-intestinal issues, blood pressure regulation, cancer prevention, weight loss, boils, wounds, fever, and ulcers [29]. Furthermore, As shown in Table 3, some of the antidiabetic activities of *P. guajava* phytochemicals include modulation of glycogen metabolism by phenolic compounds, pancreoprotective effects of quercetin, antioxidant activity of epicatechin, and hypoglycemic effects of chlorogenic acid, among others.

In animal studies, *P. guajava* was found to lower plasma glucose, cholesterol, and triglycerides [52]. In a study investigating the antihyperglycemic effects of *P. guajava* in diabetic rats, the oral administration of *P. guajava* leaf extract significantly decreased serum glucose and glycosylated hemoglobin concentrations, while also enhancing plasma insulin and hemoglobin [53]. The extract was speculated to have improved hyperglycemia by enhancing insulin sensitivity and glucose uptake or modulating glucose metabolism pathways [53]. Additionally, guava fruit had previously shown potential in protecting the kidneys against diabetic progression through its antioxidant, anti-inflammatory, and anti-glycative effects [53]. These findings highlight the nutraceutical potential of guava in managing diabetes-related complications.

*P. guajava* leaf extract exhibits various antidiabetic mechanisms, as shown in Table 4, including insulin sensitization, glucose uptake stimulation, gluconeogenesis inhibition, preservation of β cell function, incretin-based therapy, inhibition of carbohydrate digestion and absorption, and anti-inflammatory/antioxidant effects. Combination studies with other plants are essential to explore synergistic effects and enhance the overall therapeutic potential of *P. guajava*. By combining *P. guajava* with other medicinal plants, it is possible to target multiple pathways involved in diabetes pathogenesis, improve glycemic control, and potentially minimize side effects. Furthermore, combining different plant extracts may offer a broader spectrum of bioactive compounds, enabling a comprehensive approach towards diabetes management. Such combination studies have the potential to contribute to the development of more effective and holistic antidiabetic treatments.

## 4. Polyherbalism in the Management and Treatment of DM

The formulation of African traditional medicines relies heavily on polyherbal mixtures, combining multiple plants to create medicinal regimens. This approach, known as polyherbalism, resembles the polytherapy commonly seen in clinical practice. While individual plant use may not directly control serum glucose concentration effectively, they often offer indirect benefits such as antioxidant and anti-inflammatory effects compared to conventional treatments [47]. However, the combined action of various medicinal plant extracts enhances overall effectiveness due to synergistic interactions among bioactive compounds [61]. This synergy arises from interactions with different receptors in various organs, resulting in a more potent therapeutic effect. Combinations of medicinal plants may provide an additive or synergistic antidiabetic effect due to their complementary phytochemical profiles and traditional uses in managing DM [62].

**Figure 3:**
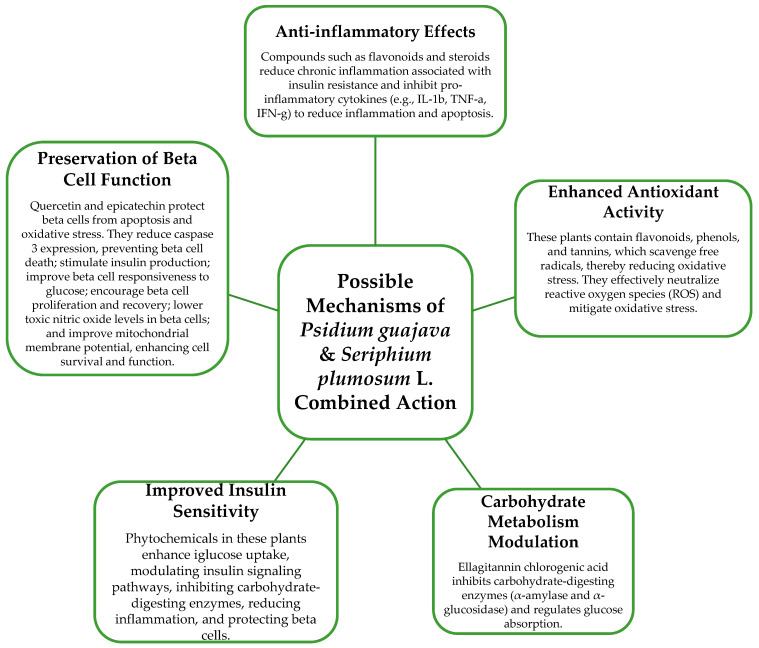
An illustration of the possible combined antidiabetic mechanisms of *P. guajava* and *S. plumosum*, showcasing potential synergistic or additive interactions.

Hypothetically, as depicted in Figure 3 below, and based on the documented properties of individual plants found in the literature, the combined antidiabetic mechanism or activity of these two plants could entail the following scenarios:

The following discussion explores the possible combined antidiabetic mechanisms of *P. guajava* and *S. plumosum*, focusing on their phytochemicals. Despite the absence of direct scientific evidence, there exists anecdotal reports from traditional health practitioners of their use in combination for diabetes management. Drawing insights from other medicinal plants used in combination, the discussion highlights the potential synergistic effects of blending medicinal plants, showcasing the efficacy of such plant combinations in traditional medicine.

Enhanced antioxidant activity: Both plants contain phytochemicals known for their antioxidant properties, such as flavonoids, phenols, and tannins. Combining these antioxidants may provide additive or synergistic protection against oxidative stress, a key factor in the pathogenesis of DM. A combination of aqueous extracts from *Spilanthes africana*, *Portulaca oleracea*, and *Sida rhombifolia*, mixed equally, exhibited significant scavenging properties on DPPH and OH radicals in STZ-induced diabetic rats (intraperitoneal 50 mg/kg b.w.), indicating a strong antioxidant effect. This plant mixture demonstrated hypoglycemic, antioxidant, and hypolipidemic properties, suggesting its potential for managing DM. The mixture solution of the three plant extracts was prepared in a 1:1:1 ratio for the antidiabetic assay. Serial dilutions (20, 40, 80, 120, and 240 μg/mL) of each extract and the mixture were used to determine in vitro antioxidant activity. This evidence supports the idea that combining medicinal plant extracts may enhance overall antioxidant activity, potentially offering increased protection against oxidative stress, a key factor in DM [63].

Improved insulin sensitivity: Phytochemicals present in both plants, such as flavonoids and phenolic compounds, have been reported to enhance insulin sensitivity. Combining these compounds may potentiate their effects, leading to better insulin action and glucose metabolism. To illustrate, a previous study revealed that a combination of *Trigonella foenum graecum* (fenugreek) seed and *Morus alba* L. (mulberry) leaf extracts inhibited α-amylase and α-glucosidase enzymes in a dose-dependent manner, with IC_50_ values of 73.2 μg/mL and 111.8 ng/mL, respectively. These botanicals also enhanced insulin sensitivity and glucose uptake in human adipocytes, prompting the development of an optimized formula. In a rat model of insulin resistance and T2DM (doses ranging from 103 to 105 mg/kg through intraperitoneal injections), alloxan was administered to the animals after they were fed a high-fat diet to induce insulin resistance and T2DM. The plant formula, containing standardized extracts of mulberry leaf, fenugreek seed, and American ginseng at doses of 42.33, 84.66, and 169.33 mg/kg BW, improved fasting serum glucose concentration and insulin resistance and impaired glucose tolerance. Furthermore, it countered the reduction in GLUT4 and PDK1 expression induced by a high-fat diet and alloxan injection in adipose tissue. These findings highlight the potential of botanical interventions to positively impact metabolic health and reduce diabetes risks [64].

Modulation of carbohydrate metabolism: Components in both plants, such as ellagitannins in *S. plumosum* and chlorogenic acid in *P. guajava*, have been shown to inhibit carbohydrate-digesting enzymes and regulate glucose absorption. Combining these compounds could lead to the more effective control of postprandial serum glucose concentration.

In the aforementioned combination study, carbohydrate metabolism was modulated by enhancing glucose regulation, improving insulin sensitivity, and increasing glucose uptake in a rat model of insulin resistance and T2DM (T2DM) through a combination of fenugreek seed, mulberry leaf extracts, and American ginseng [65]. Similarly, in another study, a combination exhibited notable hypoglycemic, antioxidant, and hypolipidemic properties in streptozotocin (STZ)-induced diabetic rats. This botanical combination significantly ameliorated glycemia, total cholesterol, triglycerides, LDL-cholesterol, malondialdehyde (MDA), aspartate aminotransferase (AST), alanine aminotransferase (ALT), and creatinine concentration, while increasing HDL-cholesterol, glutathione, and total antioxidant status (TAOS) [65]. By amalgamating these findings, it becomes evident that combining medicinal plant extracts offers synergistic benefits by targeting multiple pathways involved in carbohydrate metabolism, insulin sensitivity, oxidative stress, and lipid profiles. Consequently, such interventions hold promise in effectively managing DM and mitigating associated risks.

Preservation of beta cell function: Certain phytochemicals in *P. guajava*, such as quercetin and epicatechin, have demonstrated pancreo-protective effects. Combining these with the hypoglycemic potential of *S. plumosum* may help preserve beta cell function and improve insulin secretion. A study combined Curcuma longa (rhizome) and Piper nigrum (seeds) in a ratio of 2:1 to create an herbal extract. The plant material was air-dried, finely powdered, and then suspended in sterile distilled water for soaking to prepare the combination. The aqueous extract of *C. longa* and *P. nigrum* demonstrated protective effects against beta cell apoptosis induced by pro-inflammatory cytokines. At a concentration of 1 mg/mL, the herbal extract effectively inhibited apoptosis in a Min6 beta cell line by 1.6-fold compared to cytokine cocktail treatment. The cytokine cocktail contained interleukin-1b (IL-1b) at a concentration of 50 ng/mL, tumor necrosis factor-a (TNF-a) at 25 ng/mL, and interferon-g (IFN-g) at 25 ng/mL. Moreover, it reduced nitric oxide and ROS production in beta cells, decreased caspase 3 mRNA expression, and increased mitochondrial membrane potential, all indicative of reduced beta cell damage. These results collectively indicated that the combined herbal extract helps prevent damage to pancreatic beta cells, potentially preserving their function and viability [66].

Anti-inflammatory effects: Both plants contain compounds with anti-inflammatory properties, such as flavonoids and steroids. Combining these may help reduce chronic inflammation associated with insulin resistance and contribute to overall metabolic health. A South African study investigated the anti-inflammatory activity of special tea and its blend with bush tea. While the methanol extract of bush tea alone did not demonstrate anti-inflammatory effects at a concentration of 100 micrograms per milliliter, the blend with special tea exhibited potent anti-inflammatory activity, surpassing the positive control. This suggests a synergistic effect between the two teas. The study also noted that plants with antioxidant properties often possess anti-inflammatory capacity, which was observed in both the special tea and bush tea–special tea blends, indicating high antioxidant and anti-inflammatory activities. However, further research is recommended to confirm the anti-inflammatory properties of special tea through alternative pathways, such as the inhibition of specific enzymes and pathways like NF-kB activation [67].

### 4.1. Polyherbalism in the African Context

Polyherbalism is deeply ingrained in African traditional medicine for treating various ailments, including DM [68]. It involves using multiple herbs or plant extracts simultaneously to address the multifaceted nature of diseases and improve therapeutic outcomes [68]. Rooted in indigenous knowledge passed down through generations, polyherbal formulations are crafted based on observations of the natural environment and the complementary properties of different herbs [69]. In diabetes management, these formulations may include herbs like bitter melon, fenugreek, and cinnamon, known for their hypoglycemic and anti-diabetic properties [70].

Traditional healers and herbalists customize polyherbal remedies to suit individual patient needs, drawing from a rich cultural heritage surrounding health and wellness. Medicinal plants used in polyherbal formulations hold deep cultural significance, often intertwined with local customs, rituals, and folklore [47]. Indigenous communities possess invaluable knowledge about these plants’ therapeutic properties, accumulated through centuries of observation and experimentation [68]. This indigenous knowledge is essential for advancing our understanding of polyherbalism and realizing its full potential in managing diabetes.

Studies indicate widespread use of traditional medicinal plants in Africa, with approximately 53% of South Africans relying on traditional medicine (TM) for disease prevention and treatment [71]. The effectiveness of polyherbal formulations (PHFs) in managing diabetes is underscored by the development and evaluation of 83 PHFs containing bioactive components from 147 plant species known for their anti-diabetic properties [72]. Furthermore, research utilizing polyherbal extracts in diabetic rat models has shown positive outcomes, suggesting the potential efficacy of polyherbal tablets for diabetes management [3]. Examples like Kabasurakudineer demonstrate the synergistic effects of polyherbal formulations in treating metabolic diseases such as diabetes, emphasizing the significance of herbal remedies in addressing prevalent health issues [73]. The integration of traditional medicine into diabetes care highlights the necessity for further research and incorporation of herbal therapies into mainstream healthcare practices [74]. Recent developments in this field include the creation and evaluation of polyherbal tablets derived from medicinal plants, showing promising results in diabetic rat models. The development of stable oral dosage formulations signifies progress in this area of research, offering new avenues for diabetes management [73]. Collaboration between traditional healers, researchers, and healthcare practitioners is essential for unlocking the full benefits of polyherbalism in diabetes care while safeguarding indigenous knowledge for future generations.

To sum up, the discussion highlights the significant potential of combining medicinal plant extracts, particularly in the context of managing DM. Polyherbal formulations, akin to polytherapy in clinical practice, offer synergistic effects that surpass the efficacy of singular plant use. By combining various plant extracts, these formulations can elicit a range of beneficial effects, including enhanced antioxidant activity, improved insulin sensitivity, modulation of carbohydrate metabolism, preservation of beta cell function, and anti-inflammatory effects. These effects are attributed to the diverse bioactive compounds present in different plant extracts, acting collaboratively to target multiple pathways involved in diabetes and metabolic health. The promising outcomes observed in studies highlight the effectiveness of combined medicinal plants in effectively managing DM and reducing associated risks. However, further research is recommended to explore alternative mechanisms and confirm the therapeutic potential of combined plant extracts in diabetes management. By continuing to investigate and understand the synergistic interactions among medicinal plants, we can unlock new therapeutic avenues for improving diabetes management.

### 4.2. Limitations

The study faces several limitations. Firstly, its reliance on preclinical data restricts extrapolation to clinical settings due to the lack of robust clinical trials assessing the efficacy and safety of *S. plumosum* L. However, these data exist for *P. guajava* in diabetic patients. Secondly, the diverse phytochemical compositions of these plants may impact therapeutic consistency, necessitating standardized extraction methods and quality control measures. Thirdly, the incomplete understanding of molecular pathways and interactions impedes a comprehensive elucidation of their antidiabetic effects, underscoring the need for further research. Additionally, cultural practices and environmental factors may influence their efficacy and acceptability. Moreover, while traditional health practitioners may possess knowledge of these plants individually and in combination, there is a dearth of scientific studies combining these two plants in diabetes management. Interactions are more likely to occur when *P. guajava* is used in combination with *S. plumosum* as these medicinal plants possess similar active compounds, as demonstrated in Section 2.1.3 and Section 3.3 above.

## 5. Conclusions and Future Research Directions

The increasing global prevalence of T2DM poses significant challenges to public health systems and socioeconomic conditions. Current drug-dependent treatments often come with undesirable side effects and excessive costs, highlighting the need for alternative and cost-effective therapeutic approaches. Traditional medicinal plants, including *P. guajava* and *S. plumosum*, have shown potential in treating metabolic disorders like diabetes. However, limited in vitro studies and anecdotal evidence exist for combined plant therapies. Therefore, comprehensive in vitro and in vivo studies are essential to confirm the antidiabetic activities of these plants and explore their additive or synergistic effects. Such research can contribute to the development of more effective and holistic antidiabetic treatments, leveraging the rich floral biodiversity of Africa and addressing the growing burden of T2DM globally. According to our current knowledge, this review study appears to be unique and one-of-a-kind. It explores the antidiabetic potential of *P. guajava* and *S. plumosum*, with a possibility of highlighting various mechanisms of action and their therapeutic effects. The combination of these two plants and their specific effects on diabetes management has not been extensively studied or documented in the available literature. Therefore, this study fills a significant gap in our understanding of these plants’ potential synergistic and/or additive effects and their role in antidiabetic treatments.

The standardization of extraction methods is crucial to ensure consistent quality and potency of bioactive compounds. Determining the optimal dosage, conducting long-term safety studies, and investigating the specific mechanisms of action are essential areas of research. Comparative studies with standard medications would provide valuable insights. Addressing these future directions will contribute to a comprehensive understanding of the antidiabetic potential of *Seriphium plumosum* L. and *Psidium guajava*, guiding their integration into diabetes management strategies.

## Figures and Tables

**Table 1:** Miscellaneous and medicinal uses of *S. plumosum* L.

Miscellaneous Uses	
Making of household brooms and insect repellent	[15]
Starting of fires, nesting material for chickens, and making bedding for hikers	[14]
**Medicinal Uses**	
Management of DM	[15]
Cardiac conditions and epilepsy	[6]
Boiled leaves and stems inhaled for influenza virus	[16]
Antioxidant, antiglycation, and hypoglycaemic potential demonstrated in vitro	[14]

**Table 2:** Pathways through which active compounds from *S. plumosum* may exert their antidiabetic effects.

Antidiabetic Effect	Phytochemical Constituents	Ref.
**Antioxidant activity**	Tannins, flavonoids, and phenols are known for their strong antioxidant properties. DM is associated with increased oxidative stress, which damages cells and contributes to the progression of the disease. These compounds scavenge free radicals and reduce oxidative stress, thereby protecting against DM-related complications.	[17]
**Anti-inflammatory effects**	Chronic inflammation plays a significant role in the development and progression of DM. Flavonoids, phenols, and steroids have been shown to possess anti-inflammatory properties, which can help reduce inflammation and improve insulin sensitivity.	[18,19]
**Insulin secretion and glucose regulation**	Flavonoids and phenolic compounds, such as quercetin and resveratrol, have been reported to stimulate insulin secretion from pancreatic beta cells and enhance glucose uptake by peripheral tissues. These actions can help regulate glucose concentration and improve glycemic control in diabetic patients.	[20]
**Inhibition of carbohydrate-digesting enzymes**	Phenolic compounds, such as ellagitannins, have been reported to inhibit carbohydrate-digesting enzymes such as alpha-amylase and alpha-glucosidase. By inhibiting these digestive enzymes, the absorption of glucose from the intestines is delayed, resulting in improved glucose control.	[21]
**Improvement of insulin sensitivity**	Certain flavonoids, such as flavonols and flavanones, have been shown to enhance insulin signaling and improve insulin sensitivity. This enables the insulin sensitive tissues to respond to the stimulus of insulin more effectively, therefore maintaining normal serum glucose concentration.	[22]
**Modulation of lipid metabolism**	Steroids, including phytosterols, have been studied for their potential role in regulating lipid metabolism in experimental DM. They help reduce cholesterol concentration, improve lipid profiles, and reduce the risk of cardiovascular complications associated with DM.	[22]

**Table 3:** Some of the antidiabetic activities of *P*. *guajava* phytochemicals.

Compound	Experimental Design	Findings	Ref.
**Phenols** 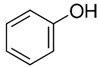	The study induced diabetes in male Sprague Dawley rats with a single 40 mg/kg intraperitoneal injection of streptozotocin and treated them with 400 mg/kg of *P. guajava* leaf extract for 14 days. Researchers examined its effects on glycogen metabolism and pancreatic protection, identifying triterpenes, phenolic compounds, and three new anti-diabetic compounds: quercetin-3-O-β-D-glucuronide, guavin B, and guavin C.	The study indicated that the antidiabetic effects of *P. guajava* might result from the modulation of glycogen metabolism mediated by phenolic compounds and triterpenes present in the extract.	[40]
**Quercetin** 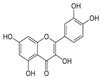	In Wistar rats with induced T2DM, quercetin concentrations ranged from 182.3 ng/mL to 244.5 ng/mL after 8 weeks of intraperitoneal administration of unspecified nicotinamide and streptozotocin, along with high fructose diets.	The combination of trehalose and quercetin has pancreoprotective and renoprotective effects against tissue injury that is induced by T2DM.	[41]
**Epicatechin** 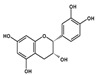	Epicatechin was administered intraperitoneally to streptozotocin (STZ)-induced diabetic rats at doses of 15 and 30 mg/kg for a period of 35 days.	Epicatechin potentially prevents T2DM complications by increased antioxidant activity and reduced oxidative stress in diabetic rats.	[42]
**Chlorogenic acid** 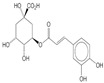	In vivo experiments in rats (HE and IHC) evaluated YBSW’s chlorogenic acids effects. In vitro assays (3-(4,5-dimethyl-2-thia-zolyl)-2,5-diphenyl-2-H-tetrazolium bromide assay, PCR, immunofluorescence, and flow cytometry) assessed apoptosis and pathways.	Chlorogenic acid B reduced triglycerides, total cholesterol, and fasting serum glucose concentrations. It increased insulin expression in islet cells, inhibited apoptosis, downregulated mRNA in the AGE-RAGE pathway, demonstrated significant hypoglycemic effects, and promoted insulin secretion while mitigating apoptosis in T2DM.	[43]
**Epigallocatechin gallate** 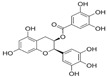	Db/db mice were orally administered with either 100 mg/kg/d by oral gavage of EGCG or GCG for 20 weeks to compare their effects on diabetic nephropathy (DN).	GCG ((−)-gallocatechin gallate) showed superior efficacy compared to EGCG ((−)-epigallocatechin-3-gallate) in treating diabetic nephropathy (DN) in db/db mice. GCG reduced water intake, urine excretion, fasting serum glucose, and systolic serum pressure and improved artery contractility, renal hemodynamics, and key renal function markers more effectively than EGCG.	[44]
**Caffeic acid** 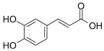	The study employed healthy and alloxan-induced type 1 diabetic (T1DM) mice. Diabetes was induced with a single intravenous injection of alloxan at a concentration of 75 mg/kg. Subsequently, caffeic acid was administered intraperitoneally for seven days at a dose of 50 mg/kg in diabetic mice.	Caffeic acid (CA) effects on diabetes and complications in mice, finding CA treatment improved liver and kidney health, lowered serum glucose, enhanced lipid profile, reduced oxidative damage, prevented pathophysiological changes, and increased lifespan in diabetic mice.	[45]
**Isoflavonoid** 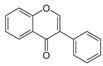	A 6-month randomized, double-blind trial with 30 postmenopausal Taiwanese women compared isoflavones to estrogen replacement therapy’s effects. One group received 100 mg isoflavone soft capsules daily, while the other received 0.625 mg conjugated estrogen daily.	Isoflavones (100 mg) and 0.625 mg conjugated estrogen equally reduced fasting serum glucose and insulin concentrations in postmenopausal women after 6 months. Fasting glucose and insulin concentrations were significantly affected by both treatments. The isoflavone group had higher serum genistein concentrations compared to the estrogen group.	[46]
**Catechin** 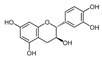	Catechin was tested for antioxidant and carbohydrate digestive enzyme inhibition in vitro. Its antidiabetic potential was assessed in Sprague Dawley rats. Different doses (2, 5, 10, 15, 30 mg/kg body weight) were given to STZ-induced diabetic rats, and effects on various parameters were evaluated.	Catechin in diabetic rats reduced serum glucose concentrations, improved body weight, and positively influenced lipid and liver function parameters. It also restored antioxidant concentrations and corrected tissue damage, indicating its potential as a diabetes treatment.	[47]
**Rutin** 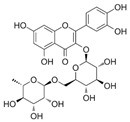	The study was a randomized controlled trial investigating rutin flavonoid effects in T2DM (T2DM) patients. Fifty T2DM patients were randomly assigned to receive either a 500 mg rutin supplement or a placebo daily for 3 months in a double-blind, placebo-controlled design.	Rutin had a significant improvement in metabolic parameters in T2DM patients who received rutin flavonoid compared to placebo. Rutin led to decreased fasting serum glucose, insulin, HbA1c, LDL cholesterol, triglycerides, and inflammatory markers, with increases in HDL cholesterol, insulin sensitivity, BDNF, and antioxidant capacity.	[48]
**Naringenin** 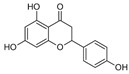	The study used 36 male albino mice (32.0–3.5 g), divided into six groups of six. The control group received standard chow; groups 2 to 6 received a high-fat diet (HFD) with 1% cholesterol. Group III also received HFD plus Naringenin (50 mg/kg).	Nar-NP administration significantly reduced body weight; glucose; insulin; leptin; cholesterol; triglycerides; and the expression of SREBP1c, pAMPK, PPAR-α, vanin-1, MCP-1, and iNOS in obese rats. Histological studies confirmed these effects, suggesting Nar-NPs as a promising obesity treatment, warranting further clinical research.	[49]
**Kaempferol** 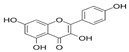	The study used C57BL6 mice. Thirty mice were divided into three groups: control (low-fat diet), HFD, and HFD + kaempferol (200 mg/kg in the diet).	Kaempferol regulates lipid metabolism; improves insulin resistance; and acts on PPAR, which improves insulin signaling. Kaempferol restores the imbalance in pancreoprotective effects against autophagy–apoptosis.	[50]
**Glycosids** 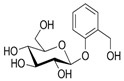	The study design involved treating HepG2 cells with test glycoside compounds at a concentration of 10 mM for 24 h, with rosiglitazone as a positive control. Specifically, HepG2 cells were exposed to compounds 2, 4, 5, 8, 12, and rosiglitazone at concentrations of 2.5, 5, 10, 20, and 40 mM for 24 h at 37 °C.	Effects that promote insulin secretion only when serum glucose is above 8.3 mmol L^−1^ were exhibited in humans, while the glucose transporter (GLUT4) was activated in adipose and skeletal muscles, therefore resulting in amplified glucose uptake.	[51]

**Table 4:** Some of the antidiabetic activities and mechanisms of *P*. *guajava*.

Antidiabetic Mechanism	Experimental Design	Findings	Ref.
Insulin Sensitization	Streptozotocin at 90 mg/kg for diabetes induction in Long Evans rat pups. Treatment groups received diabetic water control at 10 mL/kg, gliclazide at 20 mg/kg, and extract at 1.25 g/kg. Non-diabetic water control group received 10 mL/kg. All treatments were given orally for 28 days.	*P. guajava* leaf exhibits insulin-sensitizing effects, reducing serum glucose concentration by 16% and increasing serum insulin concentration. It enhances liver glycogen content, inhibits glucose absorption from the upper intestine, and reduces LDL-cholesterol concentration, promoting insulin secretion and liver glycogen synthesis and improving dyslipidemia.	[53]
Glucose Uptake Stimulation	Albino rats were induced to have diabetes using alloxan injection (200 mg/kg/bw) and divided into 8 groups. Different concentrations (200 and 250 mg/kg/bw) of guava leaf, seed, and pulp extracts were administered along with an experimental diet for 3 weeks, showing no mortality and indicating safe use. Blood samples were collected at 0, 7, 14, and 21 days for analysis.	The study demonstrated that polyphenol extracts from guava pulp exhibited potent antidiabetic effects, including improved metabolic parameters, decreased cholesterol and triglyceride concentration, and enhanced blood cell profiles in albino rats, suggesting their potential as a natural alternative to traditional diabetic medications.	[54]
Alpha Glucosidase Inhibition	The study evaluated the inhibitory effects of *P. guajava* extracts on α-glucosidase, α-amylase, and hepatic glucose-6-phosphatase activity, along with glucose uptake in C2C12 muscle cells and triglyceride accumulation in 3T3-L1 adipocyte-like cells, ensuring cell viability with non-toxic concentrations.	The study found that ethanolic extracts of *P. guajava* leaf and bark exhibited notable inhibition of α-glucosidase activity, with IC50 values of 1.0 ± 0.3 μg/mL and 0.5 ± 0.01 μg/mL, respectively. Additionally, the bark extract showed significant α-amylase inhibition with an IC50 value of 10.6 ± 0.4 μg/mL, suggesting their potential applications in managing type 2 diabetes by inhibiting enzymes involved in carbohydrate metabolism.	[55]
Inhibition of Gluconeogenesis	The study investigated the effects of *P. guajava* extract on diabetes in Wistar rats. Guava leaf extract was orally administered at doses of 100, 200, and 400 mg/kg body weight for 45 days to STZ-induced diabetic rats. The study assessed insulin concentration, glycogen, hepatic markers, gluconeogenic enzymes, OGTT fasting blood glucose concentration, lipid profile, and insulin signaling proteins.	The results showed significant improvements, particularly with the 200 mg/kg dose, including elevated insulin concentration, glycogen content, and activation of key effector molecules of the PI3K/Akt pathway. *P. guajava* leaf inhibits hepatic gluconeogenesis and promotes glycogen synthesis through the AMPK/ACC signaling pathways in streptozotocin-induced diabetic rats.	[56]
Preservation of Beta Cell Function	The study used methanol-based guava leaf extract at concentrations of 100, 200, and 400 mg/kg b.w. to counteract streptozotocin-induced diabetes in Wistar rats (180–220 g), alongside glibenclamide (600 μg/kg b.w.), showing promise in managing DM.	*P. guajava* leaf extract reduces hyperglycemia and oxidative stress, protects β cell viability, suppresses inflammation, and modulates the NF-kB signaling pathway in streptozotocin-induced diabetic rats.	[57]
Incretin-Based Therapies	In an in vitro study, *P. guajava* L. leaf extracts and flavonol-glycosides were tested for their impact on dipeptidyl-peptidase IV (DP-IV), a key enzyme in serum glucose regulation.	Ethanol extracts of *P. guajava* leaves exhibited in vitro dipeptidyl peptidase-4 (DPP-4) inhibitory activity. The ethanolic leaf extract demonstrated dose-dependent inhibition of DP-IV with an IC50 of 380 μg/mL, while individual flavonol-glycosides similarly exhibited dose-dependent inhibition. The recovery of flavonol-glycosides in gastrointestinal cells ranged from 2.3% to 5.3% over a 60 min period.	[58]
Inhibition of Carbohydrate Digestion and Absorption	The study examined ethanolic and supercritical fluid extracts of guava fruit at concentrations of 100 mg/L for solid extracts and 100 mL/L for liquid extracts. Cytotoxicity and glucose transport were assessed using Caco-2 epithelial cells (human colorectal adenocarcinoma), and gene expression was analyzed in these cells. STZ induced diabetes in the animals. Oral glucose tolerance tests were conducted on female C57BL/6N mice using guava fruit extracts at 400 mg/kg.	*P. guajava* leaf and fruit extracts have been shown to inhibit glucose transporters GLUT2 and SGLT1, resulting in reduced intestinal glucose transport both in vitro and in vivo. This inhibition contributes to the regulation of glucose absorption, potentially leading to improved glycemic control.	[58]
Anti-Inflammatory and Antioxidant Effects	*P. guajava* leaf extract affects prostaglandin endoperoxide H synthase (PGHS) isoforms. In vitro assays assessed its inhibition of cyclooxygenase and hydroperoxidase activities using recombinant PGHS isoforms. Cell-based experiments evaluated its impact on DNA synthesis rate and PGE2 synthesis in human colon carcinoma cells overexpressing PGHS-1 and PGHS-2.	*P. guajava* inhibits inflammation by targeting Prostaglandin endoperoxide H synthase (PGHS), a crucial enzyme involved in the synthesis of prostaglandins (PGs) that contribute to inflammation and carcinogenesis.	[59,60]

## Data Availability

The data supporting this literature review are from publicly available sources, which have been appropriately cited in the article. No additional data were created or analyzed in this study. The images used in this article, which have been presented in the paper, were captured by the authors and additional images captured are available upon written request.
